# Mutation analysis of the EGFR pathway genes, *EGFR, RAS, PIK3CA, BRAF,* and *AKT1*, in salivary gland adenoid cystic carcinoma

**DOI:** 10.18632/oncotarget.24818

**Published:** 2018-03-30

**Authors:** Kosuke Saida, Takayuki Murase, Mayuko Ito, Kana Fujii, Hisashi Takino, Ayako Masaki, Daisuke Kawakita, Kei Ijichi, Yuichiro Tada, Kimihide Kusafuka, Yoshiyuki Iida, Tetsuro Onitsuka, Yasushi Yatabe, Nobuhiro Hanai, Yasuhisa Hasegawa, Hitomi Shinomiya, Ken-Ichi Nibu, Kazuo Shimozato, Hiroshi Inagaki

**Affiliations:** ^1^ Department of Pathology and Molecular Diagnostics, Graduate School of Medical Sciences, Nagoya City University, Nagoya, Japan; ^2^ Department of Maxillofacial Surgery, Aichi-Gakuin University School of Dentistry, Nagoya, Japan; ^3^ Department of Otolaryngology, Head and Neck Surgery, Graduate School of Medical Sciences, Nagoya City University, Nagoya, Japan; ^4^ Department of Head and Neck Oncology and Surgery, International University of Health and Welfare Mita Hospital, Tokyo, Japan; ^5^ Pathology Division, Shizuoka Cancer Center, Nagaizumi, Shizuoka, Japan; ^6^ Department of Head and Neck Surgery, Shizuoka Cancer Center, Nagaizumi, Shizuoka, Japan; ^7^ Department of Pathology and Molecular Diagnostics, Aichi Cancer Center Hospital, Nagoya, Japan; ^8^ Department of Head and Neck Surgery, Aichi Cancer Center Hospital, Nagoya, Japan; ^9^ Department of Otolaryngology-Head and Neck Surgery, Kobe University Graduate School of Medicine, Kobe, Japan

**Keywords:** adenoid cystic carcinoma, salivary gland, EGFR pathway mutations, RAS mutations, SNaPshot assay

## Abstract

Adenoid cystic carcinoma (AdCC), one of the most common salivary gland carcinomas, usually has a fatal outcome. Epidermal growth factor receptor (EGFR) pathway gene mutations are important in predicting a patient's prognosis and estimating the efficacy of molecular therapy targeting the EGFR pathway. In this study of salivary gland AdCC (SAdCC), we looked for gene mutations in *EGFR, RAS* family (*KRAS, HRAS,* and *NRAS*), *PIK3CA, BRAF,* and *AKT1*, using a highly sensitive single-base extension multiplex assay, SNaPshot. Out of 70 cases, EGFR pathway missense mutations were found in 13 (18.6%): *RAS* mutations in 10 (14.3%), *EGFR* in one (1.4%), and *PIK3CA* in 5 (7.1%). None of the cases showed an *EGFR* deletion by direct sequencing. Concurrent gene mutations were found in three cases (4.3%). EGFR pathway mutations were significantly associated with a shorter disease-free (*p* = 0.011) and overall survival (*p* = 0.049) and *RAS* mutations were as well; (*p* = 0.010) and (*p* = 0.024), respectively. The gene fusion status as determined by a FISH assay had no significant association with mutations of the genes involved in the EGFR pathway. In conclusion, EGFR pathway mutations, especially *RAS* mutations, may be frequent in SAdCC, and associated with a poor prognosis for the patient.

## INTRODUCTION

Adenoid cystic carcinoma (AdCC), although rare accounting for less than 1% of all head and neck cancers, is one of the most common carcinomas of the salivary gland [1]. Factors that often influence survival include tumor stage, node status, patient age, tumor site, large nerve perineural invasion, and surgical margins. Standard treatment for this carcinoma is surgical resection followed by post-operative radiotherapy and/or chemotherapy. While lymph node involvement is uncommon, distant metastasis to the lung is frequent [1]. AdCC is a slow-growing tumor but the long-term prognosis is poor with the 10-year survival rate being 52–65% [2]. Previous studies have revealed that *MYB*-*NFIB* and *MYBL1*-*NFIB* fusions are major alterations in this carcinoma, accounting for approximately 50% and 10% of AdCC cases, respectively [3–7]. Gene alterations involving *MYB*, *MYBL1*, or *NFIB* genes have been considered to be among the primary events in AdCC development. In a study employing conditional *MYB*-*NFIB* mutant transgenic mice, expression of the oncogene resulted in the development of the carcinoma in nearly 30% of the animals [8, 9]. However, oncogenetic events associated with AdCC progression have not been well recognized.

Epidermal growth factor receptor (EGFR), a trans-membrane tyrosine kinase receptor, binds with its ligands (EGF and transforming growth factor alpha), and activates downstream signaling, which stimulates mitosis, leading to cell proliferation and inhibition of apoptosis [10]. It has been well documented that mutations of genes involved in the EGFR pathway play an important role in the pathogenesis and progression of various tumors [11–14]. Recently, anti-EGFR agents, including EGFR tyrosine kinase inhibitors and anti-EGFR antibodies, have been effectively used for tumor treatment [15, 16]. In pulmonary carcinomas, *EGFR* mutation is associated with Asians, females, non-smokers, and the adenocarcinoma type, and it has become evident that EGFR tyrosine kinase inhibitors are highly effective for pulmonary adenocarcinomas with *EGFR* mutations [17, 18]. RAS proteins, encoded by three ubiquitously expressed *KRAS*, *HRAS*, and *NRAS* genes, are located downstream of EGFR and are frequently mutated in human cancers [19]. These proteins are GTPases that may function as a molecular switch regulating the EGFR pathways responsible for proliferation and cell survival [12]. Aberrant RAS function is probably associated with a single mutation, typically at codon 12, 13 or 61 [20], and the mutation may favor GTP binding and result in constitutive activation of RAS [20]. The prognostic significance of *RAS* mutations has been repeatedly reported in pulmonary carcinomas [21–24]. On the other hand, it has been firmly established that anti-EGFR antibody therapy is less effective in colonic adenocarcinomas harboring mutated *RAS* genes [25]. These observations suggest that detection of EGFR pathway gene mutations is critically important not only for clarifying oncogenesis and tumor progression but also for selecting the most effective molecularly targeted therapy. Unfortunately, mutations of EGFR pathway genes in salivary gland AdCC (SAdCC) have been poorly understood.

In this study, using a large cohort of SAdCC cases, we looked for mutations of the genes involved in the EGFR pathway (*EGFR*, *RAS* family, *PIK3CA*, *BRAF*, and *AKT1*), and correlated the results with the clinicopathological features and prognostic outcomes of the patients. These genes were chosen since the mutations have been detected in some SAdCC cases by a previous comprehensive analysis using next generation sequencing (NGS) [26–29] and have been closely associated with current molecularly targeted therapy [30]. For detection of the point mutations of the above genes, we employed the SNaPshot assay, a single-base extension multiplex assay that is highly sensitive, low-cost, and rapid [31–34].

## RESULTS

### Carcinoma cases

The clinicopathological characteristics of the SAdCC cases analyzed in this study (*n* = 70) are summarized in Table 1. The tumor cases consisted of 29 men and 41 women, with ages ranging from 27 to 82 years (median, 64). In 45 cases, the tumor had originated in the major salivary glands, and in the remaining 25, in the minor salivary glands. In 28 cases, the tumor was more than 2 cm in diameter, and in 11, there was metastasis to the regional cervical lymph nodes. Twenty-nine cases were classified as stage III/IV tumors. Surgical resection was carried out in all 70 cases and neck dissection was additionally performed in 39. On pathological evaluation, 39, 13, and 18 cases were classified as grades I, II, and III, respectively. Perineural invasion was found in 42 cases. Although all tumors were resected macroscopically with curative intent, positive surgical margins were microscopically observed in 32 cases. Post-operative radiotherapy and/or chemotherapy was carried out in 30 patients but not in the remaining 40 patients because of their poor general condition and/or personal refusal. SAdCC patients with positive surgical margins tended to receive the radiotherapy. Tumor recurrence after surgery was recorded in 28 patients (median, 22.5 months; range, 1–120). At the last follow-up (median, 60.5 months; range, 7–312), 36, 27, and 7 patients were alive with no evidence of disease, alive with disease, and had died of disease, respectively. None of the patients died of other causes.

**Table 1:** Clinicopathological characteristics of the patients (*n* = 70)

Factor		*n* (%)
Age (years)	Mean	62.5
	Median	64 (range, 27–82)
	<60	25 (35.7)
	>60	45 (64.3)
Sex	Male	29 (41.4)
	Female	41 (58.6)
Primary tumor site	Parotid	17 (24.3)
	Submandibular	21 (30)
	Sublingual	7 (10)
	Minor	25 (35.7)
Tumor size (cm)	<2	42 (60)
	>2	28 (40)
Nodal status	Positive	11 (15.7)
	Negative	59 (84.3)
Clinical stage	I, II	41 (58.6)
	III, IV	29 (41.4)
Neck dissection	Performed	39 (55.7)
Post-operative radiation	Performed	30 (42.9)
Post-operative chemotherapy	Performed	3 (4.3)
Histological grade	I	39 (55.7)
	II	13 (18.6)
	III	18 (25.7)
Perineural invasion	Positive	42 (60)
	Negative	23 (32.9)
	Undetermined	5 (7.1)
Microscopic surgical margin	Positive	32 (45.7)
	Negative	38 (54.3)
Follow-up (months)	Median	60.5 (range, 7–312)
Tumor recurrence	Yes	28 (40)
Deceased	Yes	7 (10)

### Tissue FISH analysis for *MYB, MYBL1,* and *NFIB* gene translocations

We performed fluorescence *in situ* hybridization (FISH) analysis in 70 SAdCC cases using paraffin sections [7], and succeeded in obtaining FISH signals in 52 (74%) but failed to obtain the signals in the remaining 18 (26%) cases after repeated FISH procedures. Gene splits in *MYB*, *MYBL1*, and *NFIB* genes were detected in 33, 6, and 32 cases, respectively, and in 45/52 (86.5%) cases in total. In the other 7 cases, no gene splits were noted. Subsequently, we performed tissue FISH analysis for gene fusions in gene-split-positive cases. According to our previous study [7], 52 cases were divided into six gene groups; 23 *MYB*-*NFIB* (44%), 10 *MYB-X* (19%), 4 *MYBL1-NFIB* (8%), 2 *MYBL1-X* (4%), 6 *NFIB-X* (12%), and 7 (13%) cases that were completely negative (X was defined as genes whose alterations were not detected in the present FISH assay).

### Mutation analysis

**Figure 1:**
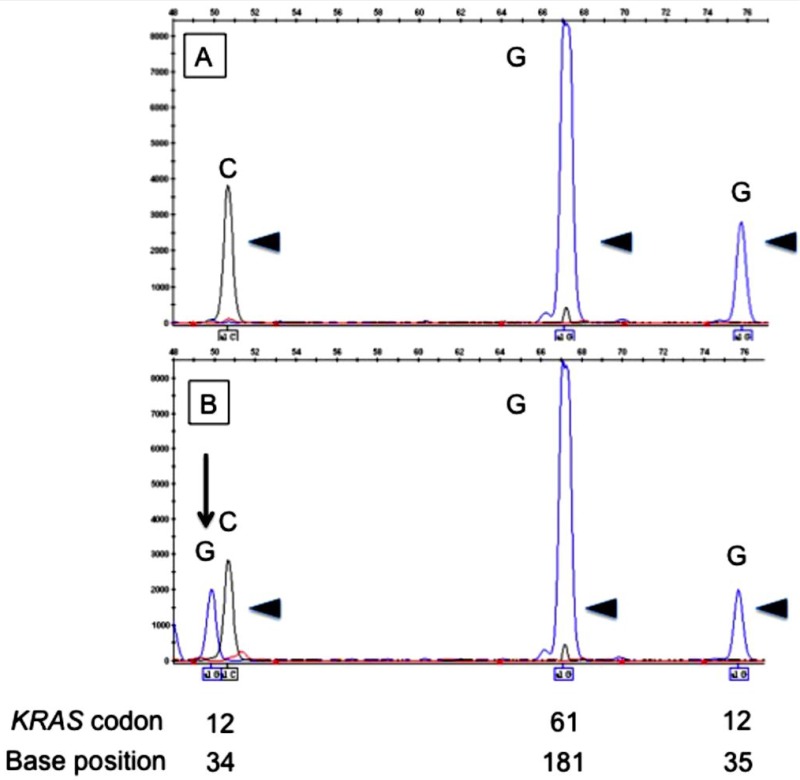
Electropherograms of the SNaPshot assay for *KRAS* codons 12 and 61 The upper panel (**A**) shows unmutated (arrowheads) codons 12 and 61. The lower panel (**B**) shows mutated codon 12 (arrow) and unmutated codon 61 (arrowheads). Positions of codons and nucleotides are indicated at the bottom of the figure.

We looked for gene mutations in *EGFR, RAS* family (*KRAS*, *HRAS*, and *NRAS*), *PIK3CA*, *BRAF*, and *AKT1*, by SNaPshot assay, a highly sensitive single-base extension multiplex assay [31–34] and EGFR deletion was detected by direct sequencing. Of the 70 SAdCC cases, 16 point mutations in the EGFR pathway were detected in 13 (18.6%) cases: *EGFR* in one (1.4%), *RAS* family mutations in 10 [14.3%; *KRAS* in 6 (8.6%), *HRAS* in 4 (5.7%), and *NRAS* in none], *PIK3CA* in 5 (7.1%), *BRAF* in none, and *AKT1* in none. All these mutations were missense mutations and none of the cases showed an *EGFR* deletion. The representative results of the SNaPshot assay are shown in Figure 1. While five point mutations were found to be positive by direct sequencing, all point mutations were validated by a SNaPshot analysis using both sense and anti-sense probes and with a polymerase chain reaction (PCR)-based amplification refractory mutation system (ARMS-PCR, a modified allele-specific PCR assay). Among the 13 cases positive for EGFR pathway mutations, tissue FISH analysis was successfully performed in 8 cases, all of which showed gene translocations associated with *MYB, MYBL1*, and *NFIB* genes. Details of the mutations and clinicopathological features of the 13 mutated cases are shown in Table 2.

**Table 2:** Salivary gland AdCC cases with gene mutations in the EGFR pathway

Case No.	Sex/Age (years)	Primary tumor site	TNM (stage)	Histologicalgrade	Perineural invasion	Microscopic margin	Gene translocation	Gene mutation	PORT	LRR	Distant metastasis	Follow-up (month)	Outcome
1	M/65	Major	T1N0M0 (I)	I	No	Negative	*MYB*-*NFIB*	*KRAS*, p.G12R	No	No	No	20	NED
2	F/51	Minor	T3N1M0 (III)	I	Yes	Positive	*MYBL1*-*X*	*KRAS*, p.G12R *PIK3CA*, p.E545K	No	Yes	Liver	60	AWD
3	M/81	Minor	T2N0M0 (II)	III	Yes	Positive	*MYB*-*X*	*HRAS*, p.Q61K	Yes	Yes	No	65	AWD
4	F/66	Major	T1N0M0 (I)	I	Yes	Negative	ND	*KRAS*, p.G12R	No	Yes	Lung	261	DOD
5	F/80	Major	T4aN0M0 (IV)	III	No	Positive	ND	*HRAS*, p.Q61K *PIK3CA*, p.E545K	Yes	Yes	Lung	7	DOD
6	M/56	Major	T3N2M0 (IV)	III	Yes	Positive	ND	*HRAS*, p.Q61K	Yes	No	Brain	24	DOD
7	F/58	Minor	T1N0M0 (I)	III	Yes	Positive	ND	*KRAS*, p.G13S	No	Yes	No	57	AWD
8	M/77	Minor	T4aN2bM0 (IV)	I	Yes	Negative	*MYBL1*-*NFIB*	*EGFR*, p.L858R *PIK3CA*, p.E545K	No	No	No	42	NED
9	M/62	Major	T2N0M0 (II)	II	No	Positive	*MYB*-*NFIB*	*KRAS*, p.G12C	Yes	No	Lung	70	AWD
10	M/72	Major	T4aN0M0 (IV)	I	Yes	Negative	*MYB*-*NFIB*	*PIK3CA*, p.E545G	No	No	No	18	NED
11	F/56	Major	T4aN0M0 (IV)	I	No	Positive	ND	*KRAS*, p.G13D	Yes	No	Lung	116	AWD
12	M/47	Major	T4aN1M0 (IV)	III	No	Positive	*MYBL1*-*X*	*HRAS*, p.Q61K	Yes	Yes	No	102	DOD
13	M/38	Major	T2N0M0 (II)	I	Yes	Negative	*MYB-X*	*PIK3CA*, p.545K	No	Yes	Yes	104	AWD

ND, not detected; PORT, post-operative radiation therapy; LRR, loco-regional recurrence; NED, no evidence of disease; AWD, alive with disease; and DOD, died of disease.

### Clinicopathological correlation and prognostic impact of gene mutations

As shown in Table 3, no correlation was found between EGFR pathway mutations and clinicopathological factors. *RAS* mutations were significantly associated with a microscopically positive margin (*p* = 0.036). Noting the case number of each gene group and taking into consideration that *MYBL1* is a member of the *MYB* family, we analyzed the clinicopathological impact of *MYB-NFIB (n = 23), MYB*-split-positive (*n* = 33), and *MYB/MYBL1*-split-positive (*n* = 39) mutations (Table 4). The *MYB-NFIB* fusion was found to have no association with the clinicopathological factors examined and the *MYB*-split-positive group was associated with a histological grade III status (*p* = 0.018). The *MYB/MYBL1*-split-positive group was associated with a histological grade III status (*p* = 0.023), a positive perineural invasion (*p* = 0.037), and a microscopically positive margin (*p* = 0.008). None of these gene groups was associated with an EGFR pathway mutation or *RAS* mutation.

**Table 3:** Association between EGFR pathway mutations and clinicopathological factors

Factor		EGFR pathway mutations		*p*	*RAS* mutations		*p*
		Positive	Negative		Positive	Negative	
Age	>60y	7	38	0.523	5	40	0.477
	<60y	6	19		5	20	
Sex	Male	8	21	0.127	5	24	0.731
	Female	5	36		5	36	
Tumor site	Major	9	36	0.759	7	38	1.00
	Minor	4	21		3	22	
Tumor size	I/II	6	36	0.349	5	37	0.507
	III/IV	7	21		5	23	
Nodal metastasis	Positive	4	7	0.198	3	8	0.186
	Negative	9	50		7	52	
Clinical stage	I/II	6	35	0.361	5	36	0.731
	III/IV	7	22		5	24	
PORT	Performed	6	24	1.00	6	24	0.308
	Not received	7	33		4	36	
Histological grade	I/II	8	44	0.296	5	47	0.111
	III	5	13		5	13	
Perineural invasion	Positive	8	34	1.00	5	37	0.710
	Negative	4	19		4	19	
Microscopic margin	Positive	8	24	0.232	8	24	**0.036**
	Negative	5	33		2	36	

PORT, post-operative radiation therapy.

**Table 4:** Association of gene alteration group with clinicopathological factors (52 cases in total)

	Gene group (*p*)		
	*MYB-NFIB*	*MYB*-split-positive	*MYB/MYBL1*-split-positive
Factor	(*n* = 23)	(*n* = 33)	(*n* = 39)
Age >60 years	0.77	0.546	0.334
Sex	1.00	0.771	0.743
Tumor site	0.77	0.382	0.177
Tumor size	0.778	0.766	0.099
Nodal metastasis	0.307	0.729	0.051
Clinical stage	0.574	0.558	0.057
PORT	0.397	0.371	0.328
Histological grade	0.524	**0.018** (grade III)	**0.023** (grade III)
Perineural invasion	0.398	0.070	**0.037** (positive)
Microscopic margin	0.400	0.149	**0.008** (positive)
EGFR pathway mutations	1.00	1.00	0.177
*RAS* mutations	1.00	1.00	0.314

PORT, post-operative radiation therapy; DFS, disease-free survival; and OS, overall survival.

**Figure 2:**
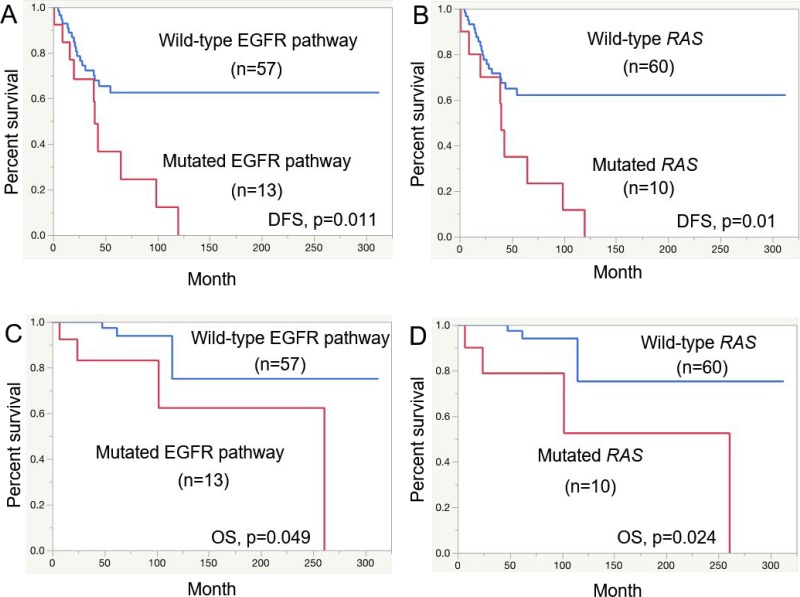
Prognostic analysis for salivary gland AdCC patients Disease-free survival (DFS) for EGFR pathway mutations (**A**) and *RAS* mutations (**B**) and overall survival (OS) for EGFR pathway mutations (**C**) and *RAS* mutations (**D**).

The disease-free survival (DFS) rates of the SAdCC patients at 5 years and 10 years were 57.9% and 31.4%, respectively, and overall survival (OS) rates at these points were 94.7% and 72.7%, respectively. Results of the prognostic analysis are shown in Table 5. With respect to DFS, T3/4 tumors (*p* = 0.006), clinical stage III/IV (*p* = 0.006) tumors, histological grade III (*p* = 0.009) tumors, tumors with a microscopically positive margin (*p* = 0.013), and those with EGFR pathway mutations (*p* = 0.011, Figure 2A) and *RAS* mutations (*p* = 0.010, Figure 2B) were all significantly associated with a shorter patient survival. For OS, histological grade III (*p <* 0.0001), microscopically positive margins (*p* = 0.023), EGFR pathway mutations (*p* = 0.049, Figure 2C), and *RAS* mutations (*p* = 0.024, Figure 2D) were selected as risk factors with statistical significance. An *MYB-NFIB, MYB*-split-positive, and *MYB/MYBL1*-split-positive status had no prognostic impact on either DFS or OS (Table 5).

**Table 5:** Prognostic analysis (70 cases in total)

Factor		*p*	
		DFS	OS
Age	>60y	0.066	0.976
Sex	Male	0.183	0.646
Tumor site	Major	0.510	0.482
Tumor size	T3/4	**0.006**	0.086
Nodal metastasis	Positive	0.267	0.057
Clinical stage	III/IV	**0.006**	0.089
Histological grade	III	**0.009**	**<0.0001**
Perineural invasion	Positive	0.146	0.869
Microscopic margin	Positive	**0.013**	**0.023**
Mutations in *EGFR* pathway	Positive	**0.011**	**0.049**
*RAS* mutations	Positive	**0.010**	**0.024**
*MYB-NFIB* (*n* = 52)	Positive	0.646	0.609
*MYB*-split (*n* = 52)	Positive	0.562	0.499
*MYB/MYBL1*-split (*n* = 52)	Positive	0.220	0.170

DFS, disease-free survival; OS, overall survival

## DISCUSSION

In examining our 70 SAdCC cases, we searched for mutations in genes involved in the EGFR pathway [*EGFR*, *RAS* family (*KRAS*, *HRAS*, and *NRAS*), *PIK3CA*, *BRAF*, and *AKT1*]. All AdCC cases included in this study were retrieved from salivary gland cases since surgical procedures [35], TNM classifications [36], and prognoses [37, 38] are different between SAdCC cases of the salivary gland and those of other head and neck sites. Of the 70 SAdCC cases, gene mutations were detected in 18.6% (*RAS* in 14.3%, *PIK3CA* in 7.1%, and *EGFR* in 1.4%) but not in *BRAF* or *AKT1*. All these mutations were missense mutations and none of the cases showed an *EGFR* deletion. Concurrent mutations were found in 4.3%. In the prognostic analysis, *EGFR* pathway mutations were selected as a risk factor for DFS (*p* = 0.011) and OS (*p* = 0.049) as were *RAS* mutations, (*p* = 0.010) and OS (*p* = 0.024), respectively. In the 70 AdCC cases, the fusion status was successfully determined in 52 using custom-made FISH probes. An *MYB-NFIB* and *MYB*-split-positive status had no or only a weak clinicopathological impact whereas an *MYB/MYBL1*-split-positive status was associated with a higher tumor grade and a local aggressiveness of the tumor but failed to have a prognostic impact.

One of the important findings of this study was that *RAS* mutations were frequently detected (14.3%) in SAdCC cases. Anti-EGFR antibodies, including cetuximab and panitumumab, are promising molecularly targeted drugs for head and neck carcinomas including AdCC. Hitre *et al*. recently reported that in a phase II trial with cetuximab combined with conventional chemotherapy and/or radiotherapy, an objective response was obtained in >40% of patients with locally advanced or metastatic head and neck AdCCs [39]. Locati *et al*. reported treating 23 patients with recurrent and/or metastatic salivary gland AdCCs with cetuximab alone and 12 of these patients remained in a stable disease state for more than six months [40]. Jacob *et al*. reported treating 18 patients with recurrent and/or metastatic salivary gland AdCCs with orally administered gefitinib and seven of these patients showed prolonged disease stabilization for more than nine months [41]. Unfortunately, the *RAS* mutation status was not examined in these trials. It is well known that colorectal adenocarcinomas with *RAS* mutations often respond poorly to anti-EGFR antibody therapy. In head and neck squamous cell carcinomas, *RAS* mutations may confer resistance to therapies using EGFR tyrosine kinase inhibitors or anti-EGFR antibodies in experimental and clinical studies [42]. It should be determined whether *RAS* mutations have a similar value in predicting efficacy in SAdCC patients. On the other hand, *RAS* genes may be important therapeutic targets. For some carcinomas, cancer vaccines that stimulate immunity against mutant RAS proteins and antisense therapies that block the translation of mutant *RAS* genes can be applied in treatment [43]. Evidence using cancer cell lines has indicated that *KRAS* antisense oligodeoxynucleotide inhibits KRAS expression, tumor growth, and tumor invasiveness [44, 45], and that the oligodeoxynucleotide can also suppress peritoneal dissemination of cancer cells *in vivo* [45, 46].

Another important finding of this study was that the *RAS* mutations were identified as risk factors for both DFS and OS. To the best of our knowledge, this finding has not been reported for SAdCC. The prognostic significance of *RAS* mutations varies according to the cancer type; and while it has not been established in colonic adenocarcinoma [47], it has been repeatedly reported in pulmonary carcinomas [21–24]. The salivary glands are developmentally close to the lungs where many salivary gland type tumors including AdCC frequently occur [48]. In addition, *RAS* mutation rates may be similar in pulmonary adenocarcinoma and SAdCC [21–24]. We speculate that the significance of *RAS* mutations in SAdCC may be similar to that in pulmonary carcinomas. Although the biological significance of *RAS* mutations in SAdCC oncogenesis remains to be elucidated, according to lung carcinoma oncogenesis [49, 50], *RAS* mutations may be a relatively late genetic event. Gene fusions involving *MYB, MYBL1*, and *NFIB*, which were found in nearly 90% of our SAdCC cases, are currently considered to be the primary oncogenetic event in SAdCC [8, 9], and *RAS* mutations may confer progressive or invasive features on the tumor cells, resulting in a worse prognosis for SAdCC patients.

The *RAS* gene mutation rate (14.3%) in our SAdCC cases was somewhat higher than those reported in previous studies (0–9.1%), as summarized in Supplementary Table 1 [26–30, 51, 52]. This difference may be explained partly in terms of the heterogeneity of the carcinomas and the detection methods employed. For detection of point mutations, we used the SNaPshot assay [31–34]. This is a highly sensitive single-base extension multiplex assay that requires less than 5% mutant alleles to identify mutations [32]. Allele-specific PCR assays (including ARMS-PCR that we used for the mutation validation) may have a sensitivity similar to that of SNaPshot while conventional direct sequencing requires more than 20% mutant alleles [53]. NGS offers simultaneous sequencing of thousands to millions of short nucleic acid sequences in a multi-parallel fashion [54, 55]. In NGS, however, the detection sensitivity is largely dependent on the depth-of-coverage and the default calling parameters for an automatic analysis because NSG has the inherent weak points of short-length amplicons and a low reliability of the sequencing data of each read. In two landscape studies of AdCC [26, 27] where the depths-of-coverage were low, *RAS* mutations were infrequent (0% and 1.7%) while in two target sequence studies of AdCC [28, 29], *RAS* mutations were frequent (6.1% and 9.1%), similar to our study (14.3%) (Supplementary Table 1). We rigorously confirmed the mutations by (1) testing all samples in at least two separate experiments, (2) validating the point mutation results with the ARMS-PCR assay, and (3) carrying out a SNaPshot assay using both sense and anti-sense probes (Supplementary Figure 1).

While gene mutation rates of *EGFR, BRAF*, and *AKT1* were low in our study, the *PIK3CA* mutation was detected in 5/70 (7.1%) SAdCC cases. These rates were similar to those of previous AdCC studies (Supplementary Table 1) [26–30, 51, 52]. Owing to the small number of mutated cases, we were unable to clarify the clinicopathological significance of the *PIK3CA* mutation in SAdCC. The prognostic impact of this mutation has not been established in other carcinomas, but constitutive activation of PI3K by *PIK3CA* mutation has been associated with resistance to trastuzumab therapy targeting HER2 [56]. However, since HER2 overexpression and *HER2* mutation are rare in SAdCC [57], the presence of a *PIK3CA* mutation may not be very important as an efficacy-predicting biomarker. Similarly, mutations in *EGFR*, *BRAF*, and *AKT1* were found to be very rare in our SAdCC cases, suggesting their limited role in the diagnosis and treatment of these cases.

As we recently described [7], we divided our SAdCC cases (*n* = 52) into six gene groups. Since the case numbers in some gene groups were small, it was difficult to draw a definite clinicopathological contour of each group. We focused our attention on the *MYB-NFIB, MYB-*split-positive, and *MYB/MYBL1*-split-positive groups. While the former two groups had no or only a weak clinicopathological impact, the latter group was associated with a higher histological grade, perineural tumor invasion, and a positive microscopic margin (Table 4). These findings were in accord with our previous finding that *MYB/MYBL1*-split-positive tumors may be locally aggressive [7]. This is difficult to explain but it is intriguing to speculate that *MYBL1* alterations may be associated with adverse features as Brayer *et al.* previously suggested [5]. Further studies are needed to clarify whether *MYBL1* gene alterations are associated with aggressive behavior in SAdCC.

On performing FISH analysis of our 70 SAdCC cases using paraffin sections, we successfully obtained FISH signals in 52 (74%) but failed to obtain these signals in the remaining 18 (26%). This failure may be partly owing to a suboptimal DNA quality of the samples and the lengths of the FISH probes used. Our FISH probes were custom-made and the lengths (range, 180 kb to 192 kb) were shorter than those of commercially available FISH probes (usually longer than 400 kb). We randomly selected four FISH-negative cases and performed a preliminary FISH assay using commercially available *ETV6*-split probes (486 kb and 632 kb in length, Zytovision, Bremerhaven, Germany) and successfully obtained FISH signals in three cases (data not shown).

In summary, we showed that *RAS* mutations were frequent (14.3%) in our SAdCC cases and that the mutations were associated with a shorter DFS and OS in these patients. These findings may be useful in developing novel therapeutic strategies against this lethal tumor. Clarification is warranted as to whether the *RAS* mutation status would be useful in predicting the efficacy of treatments targeting EGFR pathway molecules in SAdCC patients.

## MATERIALS AND METHODS

### Case selection

From the pathology files of the authors' institutions, we retrieved 70 cases previously diagnosed as SAdCC. Cases involving the sinonasal cavity, lung or other sites were not included in this study. All cases were carefully reviewed by two independent pathologists (TM and HI) according to WHO classification criteria for salivary gland tumors [1]. None of the cases showed distant metastasis at the initial treatment. The following clinicopathological factors were analyzed: age, sex, primary tumor site, tumor size, metastasis to regional lymph nodes, clinical stage, histological grade, perineural invasion, microscopic surgical margin, post-operative treatment (radiotherapy and/or chemotherapy), and follow-up. Tumors were histopathologically classified as grade I, II, or III; grade I tumors mainly showed a tubular and cribriform pattern without solid tumor components, grade II tumors were defined as cribriform with solid components of <30%, and grade III tumors, as those showing solid components of >30% [58]. This study was approved by the institutional review board of Nagoya City University and conducted in accordance with the Declaration of Helsinki.

### Tissue FISH analysis for *MYB, MYBL1,* and *NFIB* gene translocations

Using paraffin tumor sections, we performed interphase FISH analysis for the gene splits in *MYB*, *MYBL1*, and *NFIB*, as well as gene fusions between these genes when the gene splits were present. FISH procedures have been described elsewhere [7, 59] and the FISH probes that we used are shown in Supplementary Table 2. The frequencies of gene abnormalities were determined by counting >100 tumor cells. SAdCC cases known to possess gene alterations and normal parotid glands were used as positive and negative controls, respectively. Cut-off values were evaluated in 100 non-overlapping cell nuclei of 10 normal salivary gland tissues. The samples were considered positive if >10% (mean + 3SD, rounded-up) of examined nuclei showed abnormal signals.

### SNaPshot multiplex assay for point mutations

Formalin-fixed, paraffin-embedded tumor samples were cut at 4 μm, and tissue sections were deparaffinized and lightly stained with methyl green. Tumor tissues only were scraped under a dissecting microscope using a serial hematoxylin and eosin-stained section as a guide. DNA was extracted by incubating tumor tissues at 56°C overnight in digestion buffer containing proteinase K. PCR for the β-globin gene (300 bp) was performed to test the DNA quality. Primers were designed to amplify DNA fragments containing codons 746–750 and 858 for *EGFR*; codons 12, 13, and 61 for *KRAS* and *HRAS*; codons 12 and 61 for *NRAS*; codons 542, 545, and 1047 for *PIK3CA;* codon 600 for *BRAF;* and codon 17 for *AKT1* (Supplementary Table 3). The PCR products were treated with exonuclease I (Exo-I, Takara Bio, Kusatsu, Japan) and shrimp alkaline phosphatase (SAP, Takara Bio) to remove unincorporated primers and deoxynucleotide triphosphates. A single-base extension multiplex assay was performed using a SNaPshot Multiplex Kit (Applied Biosystems, Foster City, CA, USA) according to the manufacturer's instructions. The final reaction mix (10 μl) contained 3 μl of the treated PCR product, 5 μl of SNaPshot ready reaction premix containing fluorescent dideoxynucleotides (A = ddR6G, green; C = ddTAMRA, black; G = ddR110, blue; and T = ddROX, red) and probe primers. The reaction was performed for 25 cycles under stringent conditions (96°C for 10 sec, 50°C for 5 sec, and 60°C for 30 sec). An aliquot of the SNaPshot extension reaction mixture (10 μl) was then treated with SAP, followed by enzyme inactivation for 15 min at 75°C. The probe primer sequences are listed in Supplementary Table 4. The fluorescence and size of the extended products were determined by capillary electrophoresis on an ABI PRISM 3130 genetic analyzer (Applied Biosystems). Data thus obtained were analyzed using GeneMapper v4.0 (Applied Biosystems) with specific detection parameters. The point mutations detected with the SNaPshot assay were validated using (1) a SNaPshot assay using both sense and antisense probes and (2) direct sequencing or ARMS-PCR. Primers for the ARMS-PCR assay (Supplementary Table 5) were specifically designed for each of the detected point mutations. All samples were tested in at least two separate experiments.

### Deletion analysis for the EGFR gene

Tumor DNA was extracted as described above. The PCR reaction was carried out using primers (Supplementary Table 3) for amplifying DNA fragments containing exon 19 of the *EGFR* gene. The PCR products were analyzed by DNA-chip-based electrophoresis (Agilent Bioanalyzer 2100, Agilent Technologies, Santa Clara, CA). Cases suspected of a gene deletion (E746-A750del) were subjected to direct sequencing to confirm the deletions.

### Statistical analysis

Clinicopathological features were compared using Fisher's exact test or the Mann–Whitney *U*-test. Survival curves were calculated using the Kaplan-Meier method. To identify variables significantly associated with patient survival, a log-rank test was performed. A value of *p* < 0.05 in each test was regarded as statistically significant. All statistical analyses were two-tailed and performed with the statistical software JMP (ver. 10, SAS, Cary, NC, USA).

## SUPPLEMENTARY MATERIALS FIGURE AND TABLES


