# Supplementary material for: Mutation analysis of the EGFR pathway genes, EGFR, RAS, PIK3CA, BRAF, and AKT1, in salivary gland adenoid cystic carcinoma
Source: Oncotarget. 2018 Mar 30;9(24):17043–55. doi: 10.18632/oncotarget.24818 (PMC5908304; doi:10.18632/oncotarget.24818)
Supplement: Supplementary file 1 [file oncotarget-09-17043-s001.pdf]

Mutation analysis of the EGFR pathway genes, *EGFR, RAS, PIK3CA, BRAF,* and *AKT1*, in salivary gland adenoid cystic carcinoma

SUPPLEMENTARY MATERIALS

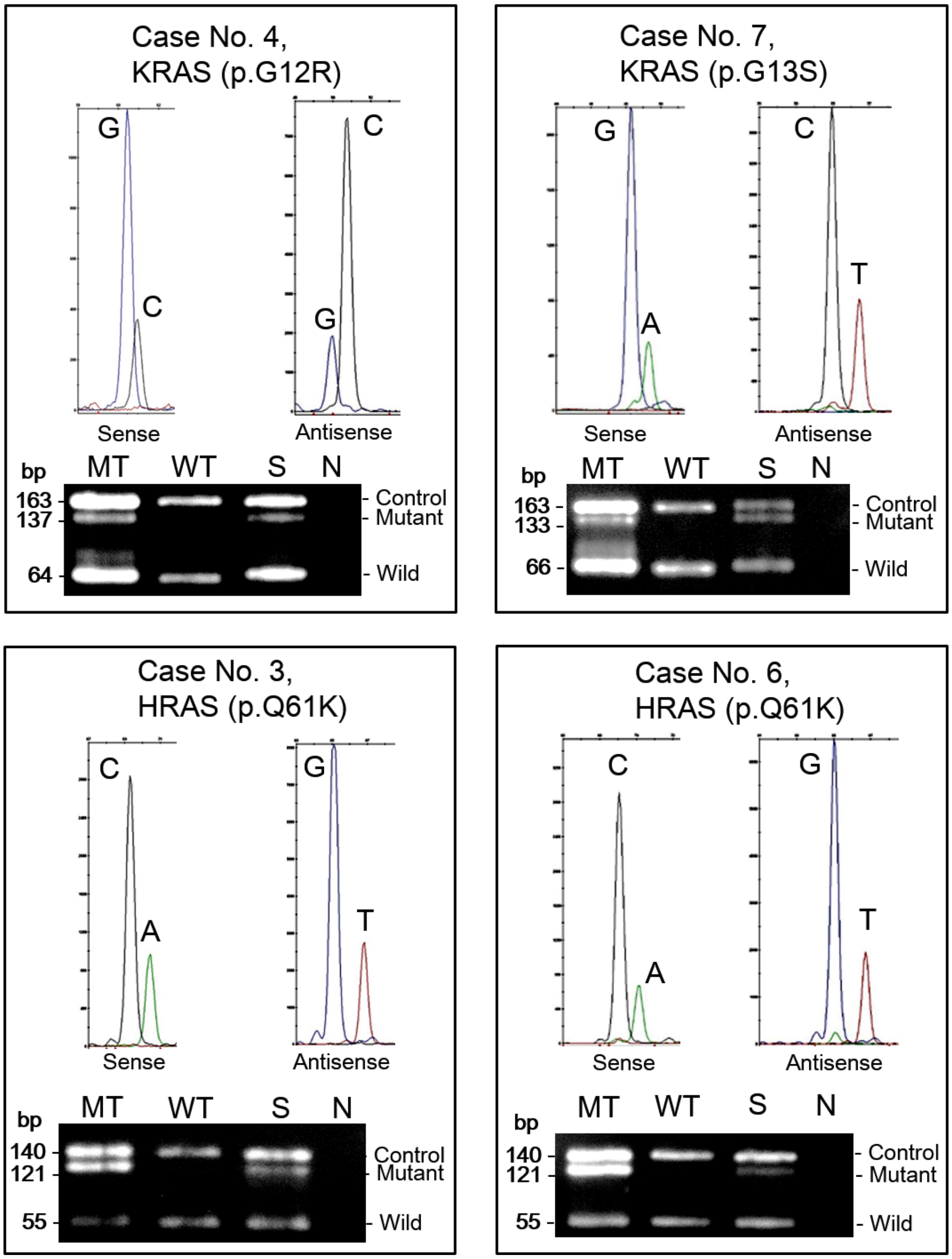

Supplementary Figure 1: Validation of SNaPshot results. The case numbers shown are correspondent with those in Table 2.

Supplementary Table 1: Mutations in the genes involved in the EGFR pathway

Authors	Dahse *et al.*	Shalmon *et al.*	Cros *et al.*	Stephens *et al.*	Ho *et al.*	Grünewald *et al.*	Kato *et al.*	Saida *et al.*
n	25	6	40	24	60	11	49	70
Primary site	Salivary	Salivary	Head & neck	Head & neck	Head & neck	Parotid	Salivary	Salivary
Assay	DS	DS	DS/AD	NGS WE: 30X	NGS WE: 100X WG: 38X	Target seq.	Target seq.	SNaPshot
RAS (%)	4	0	7.5	0	1.7	9.1	6.1	14.3
KRAS (%)	4	0	0	0	0	0	2	8.6
HRAS (%)	ND	ND	5	0	1.7	9.1	4.1	5.7
NRAS (%)	ND	ND	2.5	0	0	0	0	0
EGFR (%)	0	ND	0	0	0	0	2	1.4
PIK3CA (%)	ND	0	2.5	4.1	5	0	6.1	7.1
BRAF (%)	ND	0	2.5	0	0	0	2	0
AKT1 (%)	ND	ND	0	0	0	0	4.1	0
Reference (PMID)	51 (19362042)	52 (26811072)	30 (23933559)	26 (23778141)	27 (23685749)	28 (26053092)	29 (26247885)	Our study

DS, direct sequencing; AD, allele discrimination; NGS, next generation sequencing; and WE whole exome; WG, whole genome.

Supplementary Table 2: FISH probes

FISH	Probe	
MYB gene split	RP11-349J5	(labeled for green)
	RP11-141K5	(labeled for red)
MYBL1 gene split	RP11-346I3	(green)
	RP11-110J18	(red)
NFIB gene split	RP11-1075G4	(green)
	RP11-452J2	(red)
MYB-NFIB gene fusion	RP11-349J5	(green)
	RP11-452J2	(red)
MYBL1-NFIB gene fusion	RP11-110J18	(green)
	RP11-452J2	(red)

Supplementary Table 3: Primers used for gene amplification

Genes	Exon	Forward primer sequence (5′ to 3′)	Reverse primer sequence (5′ to 3′)	Size (base)
EGFR	19	ACCATCTCACAATTGCCA	CACACAGCAAAGCAGAAACT	170
	21	CGCAGCATGTCAAGATCACAG	TGGCTGACCTAAAGCCACCT	109
HRAS	2	CAGGAGACCCTGTAGGAGG	TCGTCCACAAAATGGTTCTG	139
	3	GGAGACGTGCCTGTTGGA	GGTGGATGTCCTCAAAAGAC	140
KRAS	2	GGCCTGCTGAAAATGACTG	GGTCCTGCACCAGTAATATG	163
	3	CCAGACTGTGTTTCTCCCTT	CACAAAGAAAGCCCTCCCCA	155
NRAS	2	GGTGTGAAATGACTGAGTAC	GGGCCTCACCTCTATGGTG	128
	3	GGTGAAACCTGTTTGTTGGA	ATACACAGAGGAAGCCTTCG	103
PIK3CA	9	AGTAACAGACTAGCTAGAGA	ATTTTAGCACTTACCTGTGAC	139
	20	GACCCTAGCCTTAGATAAAAC	GTGGAAGATCCAATCCATTT	109
BRAF	15	GACCTCACAGTAAAAATAGG	AAATAGCCTCAATTCTTACCA	121
AKT1	4	AGGGTCTGACGGGTAGAG	CCTTGTAGCCAATGAAGGT	123

Supplementary Table 4: Sequences of SNaPshot probes

Probe	Base position	Sequence (5′ to 3′)	Size (base)	Strand
EGFR				
L858R	2573	T15 CAAGATCACAGATTTTGGGC	35	Sense
HRAS				
G12C/S	34	T17 CTGGTGGTGGTGGGCGCC	35	Sense
G12D/V	35	T31 CGCACTCTTGCCCACACCG	50	Antisense
G13C/R	37	T55 CAGCGCACTCTTGCCCACAC	75	Antisense
Q61K	181	T46 CATCCTGGATACCGCCGGC	65	Sense
Q61L/R	182	T41 GCATGGCGCTGTACTCCTCC	38	Antisense
KRAS				
G12C/R/S	34	T25 GGCACTCTTGCCTACGCCAC	45	Antisense
G12A/D/V	35	T49 AACTTGTGGTAGTTGGAGCTG	70	Sense
G13D	38	T11 GTGGTAGTTGGAGCTGGTG	30	Sense
G13C/S	37	T19 CTTGTGGTAGTTGGAGCTGGT	40	Sense
Q61E	181	T41 CTCATTGCACTGTACTCCTCTT	63	Antisense
NRAS				
G12R	34	T62 CTGGTGGTGGTTGGAGCA	80	Sense
Q61L/R	182	T33 GACATACTGGATACAGCTGGAC	55	Sense
PIK3CA				
E542K	1624	T17 ACACGAGATCCTCTCTCT	35	Sense
E545K/Q	1633	T25 ATCCTCTCTCTGAAATCACT	45	Sense
E545G	1634	T21 CCTCTCTCTGAAATCACTG	40	Sense
H1047L/R	3140	T30 GAAACAAATGAATGATGCAC	50	Sense
BRAF				
V600M	1798	T27 GATTTTGGTCTAGCTACA	45	Sense
V600E	1799	T16 GATTTTGGTCTAGCTACAG	35	Sense
AKT1				
E17K	49	T15 CCACCCGCACGTCTGTAGGG	35	Sense

Supplementary Table 5: Sequences of ARMS-PCR primers and product sizes

Gene	Primer sequence (5′ to 3′)		Product size (bp)
EGFR L858R	Forward outer	CGCAGCATGTCAAGATCACAG	Control: 109<br>Wild-type: 99<br>L858R: 49
	Reverse outer	TGGCTGACCTAAAGCCACCT	
	Forward inner	CAAGATCACAGATTTTGGGCT	
	Reverse inner	GCACCCAGCAGTTTGGCCC	
HRAS Q61K	Forward outer	CAGGAGACCCTGTAGGAGG	Control: 140<br>Wild-type: 55<br>Q61K: 121
	Reverse outer	TCGTCCACAAAATGGTTCTG	
	Forward inner	ATCCTGGATACCGCCGGCA	
	Reverse inner	GGCGCTGTACTCCTCCTG	
KRAS G12R	Forward outer	GGCCTGCTGAAAATGACTG	Control: 163<br>Wild-type: 64<br>G12R: 137
	Reverse outer	GGTCCTGCACCAGTAATATG	
	Forward inner	ACTTGTGGTAGTTGGAGCTC	
	Reverse inner	ACTCTTGCCTACGCCACC	
KRAS G12C	Forward outer	GGCCTGCTGAAAATGACTG	Control: 163<br>Wild-type: 64<br>G12C: 137
	Reverse outer	GGTCCTGCACCAGTAATATG	
	Forward inner	ACTTGTGGTAGTTGGAGCTT	
	Reverse inner	ACTCTTGCCTACGCCACC	
KRAS G13D	Forward outer	GGCCTGCTGAAAATGACTG	Control: 163<br>Wild-type: 66<br>G13D: 133
	Reverse outer	GGTCCTGCACCAGTAATATG	
	Forward inner	GTGGTAGTTGGAGCTGGTGA	
	Reverse inner	GGCACTCTTGCCTACGCC	
KRAS G13S	Forward outer	GGCCTGCTGAAAATGACTG	Control: 163<br>Wild-type: 66<br>G13S: 133
	Reverse outer	GGTCCTGCACCAGTAATATG	
	Forward inner	GTGGTAGTTGGAGCTGGTG	
	Reverse inner	GGCACTCTTGCCTACGCC	
PIK3CA E545K	Forward outer	AGTAACAGACTAGCTAGAGA	Control: 139<br>Wild-type: 115<br>E545K: 66
	Reverse outer	ATTTTAGCACTTACCTGTGAC	
	Forward inner	ATCCTCTCTCTGAAATCACTA	
	Reverse inner	ATAGAAAATCTTTCTCCTGCTC	
PIK3CA E545G	Forward outer	AGTAACAGACTAGCTAGAGA	Control: 139<br>Wild-type: 116<br>E545G: 65
	Reverse outer	ATTTTAGCACTTACCTGTGAC	
	Forward inner	TCCTCTCTCTGAAATCACTGG	
	Reverse inner	CATAGAAAATCTTTCTCCTGCT	
